# Addressing Differential Attainment Among International Medical Graduates

**DOI:** 10.1192/bjo.2025.10253

**Published:** 2025-06-20

**Authors:** Taha Anjum, Subha Thiyagesh, Rajarshi Das

**Affiliations:** South West Yorkshire NHS Foundation Trust, Wakefield, United Kingdom

## Abstract

**Aims:** The aim of this project was to gain a better understanding of clinical and professional areas that international medical graduates find difficult and often these areas can be a barrier in further career progression. This project was undertaken as part of the Leadership and Management Fellow Scheme organised by the Royal College of Psychiatrists. The idea was to align the learnings to broader goals at organisational level.

**Methods:** A survey questionnaire was created to gather information about the areas that international medical graduates struggle with in their professional career. In response to this a 'Resource Hub’ was created to provide concise information on clinically relevant topics and this was made available for all participants to access. Feedback was obtained about the utility of those resources and this was used to further broaden the 'Resource Hub'.

**Results:** The areas where people struggled to gain competence ranged from membership exams (22%) to research and development (35%), mental health legislations, and trust protocols (50%).

Following this a list of topics were brainstormed and relevant resources were added on the Trust Home page.

Feedback result for the resources was collated in terms of content (84%), precision (90%), relevance (86%), and overall effectiveness (85%). Other suggestions were taken into consideration for additional resource linking to promote communication skills, tribunal reports, and preparation for appraisal.

**Conclusion:** It was a valuable project which has helped to shed light on how to facilitate provision of clinically relevant resources that would help international medical graduates in their progress and overall attainment of career goals. The recommendations have been shared across the Trust with quality improvement and medical workforce race equality standards team.

